# A Manual of Organic Materia Medica

**Published:** 1887-12

**Authors:** 


**A Manual of Organic Materia Medica.** By John M. Maisch, Phar. D. Third edition; with 257 illustrations. pp. 532. 12mo. Philadelphia: Lea Brothers & Co.

This is an excellent manual for the use of pharmaceutical students and physicians who care to become acquainted with the sources of our materia medica. All of the drugs of vegetable and animal origin now in use, whether they have found a place in the *United States Pharmacopœia* or not, are discussed, their origin, habitat, description and properties being given.

